# Decision making on organ donation: the dilemmas of relatives of potential brain dead donors

**DOI:** 10.1186/s12910-015-0057-1

**Published:** 2015-09-17

**Authors:** Jack de Groot, Maria van Hoek, Cornelia Hoedemaekers, Andries Hoitsma, Wim Smeets, Myrra Vernooij-Dassen, Evert van Leeuwen

**Affiliations:** Radboud Institute for Health Sciences, Radboud university medical center, Nijmegen, The Netherlands; Department of Spiritual and Pastoral Care 20, Radboud university medical center, PO Box 9101, 6500 HB Nijmegen, The Netherlands; Department of Intensive Care Medicine, Radboud university medical center, Nijmegen, The Netherlands; Department of Nephrology, Radboud university medical center, Nijmegen, The Netherlands; Kalorama Foundation, Nijmegen, The Netherlands

## Abstract

**Background:**

This article is part of a study to gain insight into the decision-making process by looking at the views of the relatives of potential brain dead donors. Alongside a literature review, focus interviews were held with healthcare professionals about their role in the request and decision-making process when post-mortal donation is at stake. This article describes the perspectives of the relatives.

**Methods:**

A content-analysis of 22 semi-structured in-depth interviews with relatives involved in an organ donation decision.

**Results:**

Three themes were identified: ‘*conditions*’, *‘ethical considerations’* and *‘look back’*. Conditions were: ‘sense of urgency’, ‘incompetence to decide’ and ‘agreement between relatives’. Ethical considerations result in a dilemma for non-donor families: aiding people or protecting the deceased’s body, especially when they do not know his/her preference. Donor families respect the deceased’s last will, generally confirmed in the National Donor Register. Looking back, the majority of non-donor families resolved their dilemma by justifying their decision with external arguments (lack of time, information etc.). Some non-donor families would like to be supported during decision-making.

**Discussion:**

The discrepancy between general willingness to donate and the actual refusal of a donation request can be explained by multiple factors, with a cumulative effect. Firstly, half of the participants (most non-donor families) stated that they felt that they were not competent to decide in such a crisis and they seem to struggle with utilitarian considerations against their wish to protect the body. Secondly, non-donor families refused telling that they did not know the deceased’s wishes or contesting posthumous autonomy of the eligible. Thirdly, the findings emphasise the importance of Donor Registration, because it seems to prevent dilemmas in decision-making, at least for donor families.

**Conclusion:**

Discrepancies between willingness to consent to donate and refusal at the bedside can be attributed to an unresolved dilemma: aiding people or protect the body of the deceased. Non-donor families felt incompetent to decide. They refused consent for donation, since their deceased had not given any directive. When ethical considerations do not lead to an unambiguous answer, situational factors were pivotal. Relatives of unregistered eligible donors are more prone to unstable decisions. To overcome ambivalence, coaching during decision-making is worth investigation.

## Background

In the Netherlands, relatives of potential brain dead donors must give their consent to effectuate organ donation. A majority of the Dutch population state that they are willing to be a donor [1]. Yet, only 44 % of all adults have registered in the National Donor Register. Of those registered, 61 % declare a willingness to consent to donation, 27 % refuse consent for donation and 12 % leave the decision to their relatives or an appointed person [2]. When their deceased had registered as a donor in the National Donor Register, nearly all Dutch relatives (94 %) followed the wish of the potential donor [3]. In cases, in which the deceased did not register, or had registered that the decision was to be left to his relatives, the relatives have complete authority to decide. These cases account for 75 % of all deceased qualifying for donation. In these cases, 67 % of the relatives refuse donation on behalf of the deceased potential donor [3]. This presents a large contrast with the general willingness of the majority of the Dutch population to donate.

Research suggests that the difference between general willingness and the actual decisions made by relatives might be caused by a collision of the values of relatives with those of potential donors [4], and therefore present relatives with a dilemma. It has also been suggested that relatives refuse to give consent, because they do not want to be involved in the donation procedure [5] and, because of a lack of competence to decide [6], as relatives are overwhelmed by emotions preventing them from thinking clearly, understanding information and asking pertinent questions [7, 8].

Research has also demonstrated that relatives often regret their decision afterwards, especially when they refused consent for donation [9–13].

The aim of this study was to gain insight into the decision-making process by looking at the views of relatives of potential brain dead donors. The ethical considerations i.e. the values, motives and convictions of relatives (as well as of potential organ donors) that were expressed to reach the best possible decision were explored, as well as if relatives regretted their decision and if they would have liked to have received some support during the decision-making process after the donation request. Offering support in clarifying values is not standard procedure, and it is not known if relatives would appreciate such an offer. This issue was also explored.

## Methods

### Research design

A qualitative study was conducted by interviewing relatives of 12 cases, in which consent for donation was given, and nine cases (ten interviews, because of separate talks with divorced parents of a child), in which consent for organ donation was refused. A semi-structured interview guide was developed (Table 1) for the face-to-face interviews. Topics for the interview were derived from the research aim, the review of the literature [14] and the authors’ and others’ [15] experience in the field of organ donation. Ethical categories were explicitly involved.

**Table 1:** Topics for the interviews

nr	Interview topics	Conceptual background
	Introduction: the process before the request for donation; experiences of the proxies in the hospital	
1	Considerations to decide for donation on behalf of the potential donor	Integrity, non-maleficence
2	The wishes of the potential donor concerning donation; the Dutch donor register	Patient autonomy, self-determination
3	The wishes and opinions of the participant concerning donation (by themselves)	Beneficence, justice, easy rescue, gift or sacrifice, solidarity, altruism
4	Need for coaching or (moral) counselling during the decision making process; wishes concerning the profile of the counsellor	Vulnerability, crisis, moral distress
5	Review of the decision: peace of mind	Dignity, respecting the deceased, pride in the decision
	Additional comments and evaluation of the interview	

### Recruitment period and procedure

The participants were proxies of potential donors from the Radboud university medical center in Nijmegen, the Netherlands, between 1^st^ October 2008 and 30^th^ September 2012 and from the Sint Elisabeth Hospital in Tilburg, the Netherlands, between 1^st^ October 2010 and 30^th^ September 2012. Only relatives engaged in decision-making on post-mortal organ donation were included. By purposive sampling, the number of relatives who gave consent for donation was similar to the number of relatives who refused consent for donation.

Relatives gave consent to the treating physician of the potential organ donor or to one of the transplant coordinators to disclose their address to the Primary Researcher. Consent for disclosing was granted in 52 cases. Relatives were asked by letter for an in-depth interview regarding their experiences with the donation request. Interviews were held with 24 participants, who provided written consent to be interviewed. One interviewee was excluded, because he had withdrawn from decision-making and a second was excluded, because they had thought that they had granted consent for donation, whilst the physician had understood that they had refused consent.

The 22 remaining interviews were held with ten families (17 participants), who refused to consent to donation, nine families (14 participants), who gave full consent to donation and three families (eight participants), who did not give permission for donation after brain death (DBD), but only for donation after circulatory death (DCD), whilst DBD was possible (Table 2).

**Table 2:** Participants and their relatives

Study code of eligible donor (N = 21)	Sex/age	Days in hospital	Critical injury/illness	Duration interview (h.min)	Study code of participants N = 41	Relation to (potential) donor	Sex/age	Type donation
P04	M 64	9	hemorrhage	0.56	R07	daughter	F 31	none
P22	F 4	1	Oxygen deficiency	1.07	R19	father	M 35	none
				0.58	R20	mother	F 32	none
P23	M59	13	hemorrhage	0.55	R21	sister	F55	none
					R22	sister	F55	
P31	F45	3	hemorrhage	1.02	R23	mother	F72	none
					R24	sister	F48	
P32	M52	1	hemorrhage	1.17	R25	spouse	F 50	none
					R26	son	M 18	
					R27	daughter - letter	F ?	
P34	M39	5	hemorrhage	1.30	R28	spouse	F 34	none
					R29	friend	F 34	
P42	M46	1	hemorrhage	1.23	R32	spouse	F 47	none
P45	M26	5	Head injury (car accident)	0.49	R33	spouse	F 21	none
					R34	mother in law	F 54	
					R35	father in law	M 52	
P49	F45	12	hemorrhage	1.05	R41	sister	F 51	none
					R42	brother in law	M 51	
P01	M 54	8	hemorrhage	1.07	R01	sister	F 53	DBD
P02	M 58	10	hemorrhage	1.09	R02	spouse	F 52	DBD
					R03	son	M 27	
					R04	daughter	F 24	
P03	F 22	1	Head injury (car accident)	1.37	R05	father	M 53	DBD
					R06	mother	F 50	
P05	M 43	16	hemorrhage	0.53	R08	partner	F 52	DBD
P08	F 57	4	hemorrhage	0.48	R09	spouse	M 55	DBD
					R10	son	M 22	
P09	F 71	6	aneurism	0.45	R11	spouse	M 77	DBD
P12	M 25	8	Head injury (car accident)	1.28	R15	mother	F 50	DBD ➜ f^a^
					R16	partner	F 22	
P17	M 59	8	hemorrhage	1.02	R18	spouse	F 56	DBD
P15	M 62	42	aneurism	1.39	R17	spouse	F 59	DCD ➜ f^b^
P11	M 44	13	head injury (bike accident)	0.43	R13	spouse	F 44	DCD
					R14	brother in law	M 49	
P38	M64	1	hemorrhage	1.30	R30	spouse	F 58	DCD
					R31	daughter	F 28	
P48	M56	2	hemorrhage	1.38	R37	spouse	F 57	DCD
					R38	son	M 26	
					R39	son	M 25	
					R40	daughter	F 23	

^a^Permission was given for DBD, but procedure failed because of sepsis of organs

^b^Permission was given for DBD, but patient did not become brain dead and DCD procedure took too much time

Permission for the recruitment procedures was obtained from the relevant Research Ethics Committee of both hospitals.

### Data collection and measurement

All of the in-depth interviews [16] were carried out by the Primary Researcher, who is an experienced pastoral counsellor. All participants were visited at home, on average, within three months following the death of their family member. Each interview lasted between 43 and 99 min (mean = 69 min). All interviews were recorded by a voice recorder and transcribed by a Secretary. The transcripts were checked by two Researchers. A summary of the transcripts based on the topics of the interview guide (Table 1) was approved by the participants.

### Analysis

**Fig. 1:**
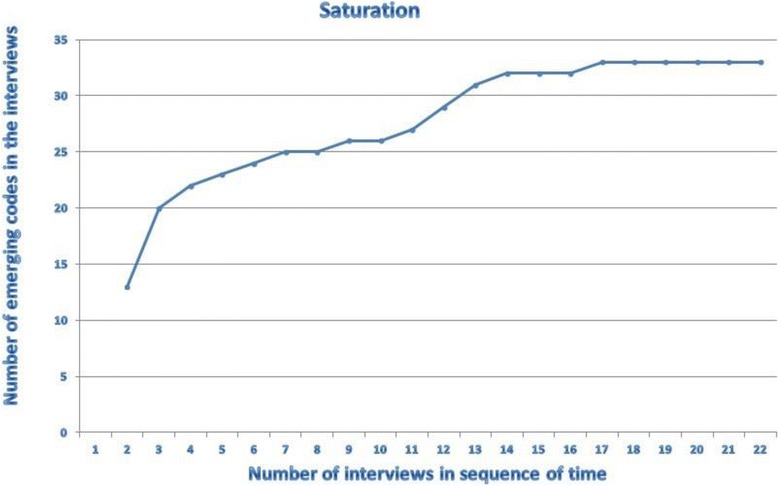
Saturation of codes in the interviews

The first three interviews were coded by two Researchers. They compared their results, and the Primary Researcher subsequently designed a codebook in cooperation with an Ethicist. With this code book, all other interviews were analysed by two Researchers using Atlas.ti 6.2.28©. The Ethicist checked their codes by sample. Consensus was reached on the attribution of the codes to the quotations. No new codes emerged after the 17^th^ interview, thus saturation [17] was reached (Fig. 1). Finally, codes were concentrated in categories and combined to themes related to the original research questions.

## Results and discussion

**Fig. 2:**
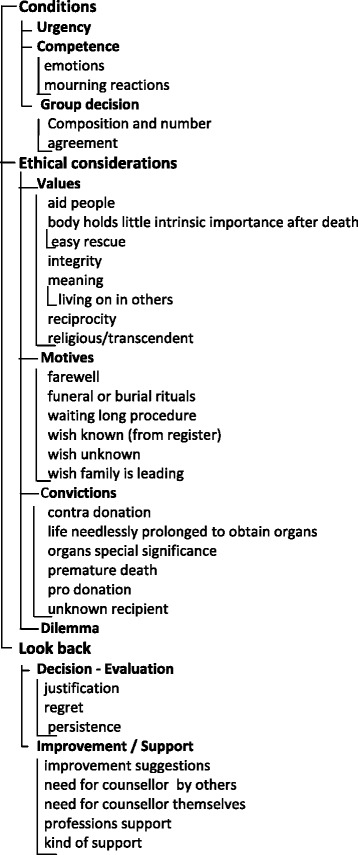
Code tree

Thirty-three codes were identified, divided (in bold text) into nine categories, and resulted in three themes (Fig. 2; Table 3).

**Table 3:** Code book – with definitions

Theme	Category	Code interview	Definition
Conditions	Urgency	Urgency	Participant mentions that s/he had little time to decide on donation
	Competence	Emotions	First reaction of participant on donation question, or participant mentions being overwhelmed by emotions, or participant mentions emotions due to the illness of deceased before the request was made
		Mourning reactions	Participant mentions mourning reactions such as being unable to understand information, not accepting the death etc.
	Group Decision	Composition and number	Number of relatives, and which relatives, were present when the donation request was made
		Agreement	Whether the relatives reached agreement on donation or not, and how they discussed it to reach agreement
Ethical considerations	Values	Aid people	Aid people, save people, give someone a better life
		Body holds little intrinsic importance after death	Utilitarian view of the body, believing that the body gives physical form to the self but is not an integral component of the self-identity
		easy rescue	He does not need his organs when he is dead, easy to give them to someone who does need them
		Integrity	Integrity of the body, protection of the body, no cutting in the body, keeping it whole
		Meaning	Donation gives at least some meaning to death, gives comfort to relatives
		live on	A part of the deceased one lives on in someone else
		Reciprocity	Indirect reciprocity refers to the notion that an individual is duty bound to help others as they themselves would want to be helped
		Religious/transcendent	Ideas on life after death, religious values on life and death
	Motives	Farewell	It is more difficult to say goodbye for relatives when they cannot be present at the moment of visible death, a reason to choose for DCD instead of DBD. And it can take a long time for the eligible donor to become officially brain dead, prolonging the farewell
		Funeral or burial rituals	The organ removal leaves marks on the body, the procedure interferes with funeral or burial rituals
		Waiting long procedure	The donation procedure takes time when the family is waiting, it is a formal and technical procedure in times of grief. Or: there is more time for family to arrive and say farewell, accept the death of their beloved
		Wish known (from register)	It was his/her last wish so we should follow it. Registration in the donor register is the main reason for consent to donation
		Wish unknown	Relatives do not know what the deceased would have wanted, he was not registered, relatives do not want to decide for someone else
		Wish family leading	Participant leaves the decision to the relatives: ‘they have to live with it’
	Convictions	Contra donation	Statements against donation in general and/or for participant himself without further motivation. And: no donation at all, also not receiving an organ himself
		Life needlessly prolonged	The life of the patient is needlessly prolonged to obtain organs, he has suffered enough
		Organs special significance	Participant makes an exception for organs with a special significance for him like the heart, eyes or skin
		Premature death	Participant mistrusts the doctors, he thinks they will not treat him well if he were registered as a donor or that organs are removed before death
		Pro donation	Statements in favour of donation in general and/or for participant himself without further motivation. For example: everyone should be registered as a donor
		Unknown recipient	Relatives do not want to donate since they do not know the recipient, his lifestyle and they cannot contact him.
	Dilemma	Dilemma	Participant mentions different motives pro and contra donation which conflict with each other and balances them, or cannot make a decision, or remains ambivalent
Look back	Decision - Evaluation	Justification	Participant explains how the decision was made, which considerations were taken into account and the way the decision was justified afterwards
		Regret	Participant mentions that he does (not) regret the decision and/or that he is proud of the decision and the way it was established. Whether the decision does justice to the wish of the deceased
		Persistence (stability of the decision)	Participant states that the decision to donate could have been different (without regretting the decision made)
	Improvement/support	Improvement suggestions	Participant mentions improvements: they needed more information, more time to deliberate with others, more (empathic) support from HCP etc.; they did not know who to ask the question to
		Need for counsellor others	Participant mentions that he can imagine that other people might need counselling, or that he might have needed it if the situation was different (e.g. if they had not known de deceased’s wish, if the relatives had not agreed etc.)
		Need for counsellor own	Participant mentions that he would (not) have wanted counselling himself, or that he asked for support
		Professions support	Which profession should give which kind of support; whether they had/should have different roles in guiding the donation procedure: physicians, nurses, transplant coordinators, social workers, psychologists, hospital chaplains. Also: whether support from the latter profession was asked
		Kind of support	Tasks that the person who supports should have, such as: giving information, mediating between family members, creating time and space to think about the question, being available all the time

The first theme concerned the conditions for decision-making after the donation request, the second theme related to the ethical considerations in decision-making and justification of the decision. The third theme concerned the look back at the decision and the decision-making process. Illustrative quotes for the categories are presented in italics.

The term ‘participants’ refers to the study participants and ‘relatives’ to the whole group (study participants and other persons engaged in the decision-making process).

### General observations

Since most interviews were planned relatively shortly after the death of their family member (median 85 days), many of the participants were in the process of grieving. They explained how their lives were turned upside down by the sudden death of their partner, child, parent or sibling. Participants frequently had some kind of re-experience of the days in the hospital environment, when talking about the event, and sometimes, details of the elapsed time were vague. Some of the participants explicitly told us that the interview had a healing function and turned out to help them cope with the tragic event.

### Theme 1: conditions for decision making by relatives of potential brain dead donors

The decision-making process was described by the relatives of potential brain dead donors, as complex, primarily because relatives had to make a decision on behalf of the deceased (surrogate decision). Three conditions contributing to this complexity were mentioned: [1] the time limit to make the decision created a sense of **urgency**; [2] the consent for donation request was made immediately after the relative had heard that a beloved one had died or was expected to die of brain death, making it difficult to focus on the request, because relatives were grieving. Half of the participants (most relatives who refused consent for donation = non-donor families) said that they were **not competent** to decide in such a crisis. “*The problem is that, often when the physician asks something, although you consciously hear the question, do you actually digest the information coming in? Because you are preoccupied by other things, you are dealing with grief or just….” (R03).* [3] The decision had to be agreed upon by a **group of relatives**. Initial disagreement between relatives –which occurred within both groups – was always overcome; agreement between relatives was mentioned as *conditio sine qua non* by the participants of both groups.

### Theme 2: Ethical considerations

#### Considerations of relatives as well as those of the potential organ donor - overview

In describing their decision-making process and providing justification for the final decision (‘yes’, ‘no’, or ‘yes with restrictions’), the participants mentioned different values, motives and convictions – their own and those of the potential donor. Specific considerations can play a very important role in the deliberation of non-donor families, whereas they were irrelevant for relatives who consented to donation (= donor families) and *vice versa*.

**Values** are used to justify the decision. The values ‘*aiding other people’* or ‘*giving people a better life*’, ‘*small effort, great benefit’* (easy rescue), ‘*reciprocity*’ or ‘*solidarity*’ were mentioned by all participants, whereas ‘*integrity*’ was important for non-donor families only. “*We were 99.9 % sure that he did not want that [donation]. Despite our idea that you should help people when you can, it’s still his body*”. *(R07) ‘Living on in other people’* was seen as a specific consideration to give meaning to organ donation. *“We are very proud of her, because she saved the life of three people. This gives me a feeling of support…. she is not really dead. A part of her lives on in someone else.” (R06)* Donor families referred to this value sometimes as a kind of comfort or relief in their grief. Participants rarely connected their mentioned values to religious views or spirituality.

**Motives** signify readiness or reluctance to act. Motives were mostly used as a practical objection contra donation: non-donor families said that it was difficult to decide on behalf of another person or words as: “*If he had really wanted organ donation, he would certainly have registered”. (R33).* Other motives that were mentioned: the donation procedure takes too long; one cannot be present at the moment of visible death; and organ donation interferes with funeral or burial rituals.

**Convictions** signify the authenticity of the decision, but may lack rationality or evidence; they are mostly expressed without any motivation *pro* or *contra* organ donation: “*Actually, I have to say that, when I turned 18, I received that form [=document for registration,* JdG*], and I did not have to think too much about it. I just signed it [opted for donation,* JdG*], because I think it is a normal thing to do”. (R16).* Most convictions lead to a refusal of consent for donation: ‘*organ donation is a reason for premature death*’; ‘*life is needlessly prolonged to obtain organs’* or ‘*the potential organ donor has suffered enough*’. Some families refused consent for donation, because of the anonymity of the recipient.

Reviewing all ethical considerations, donor families sometimes mentioned that the request to consent to donation placed them in a **dilemma**, which they could easily resolve, whereas many non-donor families held ambivalent feelings: “*I would really love to help people, and I know for sure that my Dad would have wanted that too. But it would be another major blow to let Dad fight for so long and then the organ went to someone, who did not deserve it, in his opinion, or contact [with the recipient] was not possible and we cannot see what good things Dad could still do”. (R27)*.

In balancing all ethical considerations, donor families and non-donor families came to different decisions, by giving prevalence to other values, motives or convictions. Rather remarkable is the fact that most donor families endorsed a utilitarian approach of organ donation: they see body parts as worthless after death, so they can easily give them away to someone who needs them (‘easy rescue’). Donor families emphasise ‘*aiding other people’*, ‘*giving them a better life’* or’*reciprocity*’ as important values in the field of organ donation. Organ donation offers a kind of comfort to them, because it gives meaning to an unexpected death. “*I notice then that it even feels like a kind of comfort. I am not only mourning the loss of my Father, but I also know that he has done a very good deed. I can talk about that with pride, even though my Father has just died. And I feel that to be a tremendous help in the mourning process”. (R38)*. Non-donor families, on the other hand, emphasise the integrity of the body, both from their own perspective and from that of the eligible donor. They experienced the need to protect the body of the deceased, often combined with the conviction that they have the right to make decisions concerning the dead body.

#### Specific considerations endorsing refusal of consent or consent for DCD instead of DBD

Motives mentioned by non-donor families endorsing refusal for consent were also recognised by donor families. Half of the donor families affirmed that the organ donation procedure took too long. However, for donor families the duration was not, by itself, the reason for refusal of consent; a few of the donor families even saw the extra time as an advantage. For one non-donor family though, the long procedure (combined with the anger about the utilitarian approach of the physician) was the compelling reason behind withdrawal of their initial consent: *“At a quarter to eight, we said…we’re quitting. That was when we heard that there was no longer a coughing-reflex and that they could start the procedure, which could last one and a half day, or maybe two or three and a half days. That was when we decided to pull out.”(R25)*. The long donation procedure was the primary reason for three out of the twelve donor families to agree with a DCD-procedure, although a DBD was possible, next to their wish to be present at the moment of visible death.

Both donor families and non-donor families mentioned the special significance of some organs and tissues, especially those of the heart, skin and eyes: “*NN felt that the heart had beaten only for his Father, so it should not be reserved for transplantation.” (R37).* For non-donor families, the removal of these organs was an extra consideration for refusal of consent. For a few donor families the removal of the heart was the main reason to choose for a DCD procedure, rather than a DBD.

#### Whose opinion prevails?

Important for the decision was (a) the opinion of the eligible donor on organ donation (relatives’ knowledge of that opinion and registration in the register), (b) the opinion of the relatives themselves on organ donation (either registered or non registered in the national donor register) and (c) the opinion of the participants who must decide about the body or the organs of the deceased: the deceased himself, the relatives or the physician.

The interviewed donor families and non-donor families differed on all three points. a) It was known from all but one eligible donor that they were positive about organ donation; the majority had registered as a donor. On the other hand, none of the non-donors had registered. Their opinion on organ donation was mostly unknown; if known their opinions were both in favour of, and against organ donation. b) Only some participants of the non-donor families were positive about organ donation (a few had registered), whereas most donor families were in favour of organ donation (the majority had registered). c) Remarkably, the majority of the non-donor families felt they had more right to decide about donation than the deceased, because they had to live on with the decision, whereas all donor families greatly valued the last will of the deceased.

### Theme 3: Look back

#### The **decision - evaluation**

Most participants could **justify** their decision for themselves afterwards. Although nearly all participants explicitly stated that they did **not regret** their decision, half of the non-donor families did **not persist** in their decision and explained to remain **ambivalent** on their decision, especially non-donor families, who had experienced the decision as a dilemma. Most of them disclosed that they were incapable of making a well-considered decision and continued to feel ambivalent in weighing their own values against the potential organ donors’ values or weighing the interests of the potential organ donor against those of the people on the waiting list. They thought that they might possibly have given consent if: they had more time; were prepared better; were told in advance that the procedure takes so much time; the donation request would have been posed in a more empathic and less technical way; or if physicians would have emphasised that donation can save other lives. One donor family told that their deceased would not have registered, if he had known what the procedure entails.

#### Improvement of the decision process/possibilities of supporting relatives

Non-donor families came up with more **suggestions** for improvement of the decision-making process than donor families. Flaws that were most often mentioned included: ‘*lack of information’*; followed by ‘*a short period of time to decide’*: “*Yes, and that happened in one conversation, in like, five minutes. So I think, well, how can I decide so quickly about that?*” *(R25).* Both donor families and non-donor families stated that the public information about the length of the donation procedure was not clear.

When asked whether they would have appreciated a kind of **support** or counselling around the decision making process, half of the non-donor families gave an affirmative answer, whereas the majority of the donor families would have declined such an offer. Those who would have appreciated support wanted someone like a coach or a buddy to be nearby during the whole stay in the hospital: *“When I look back, I would have found it quite nice if they had said like… well, we have a counsellor whom you have met before. He will come too and talk everything through, because these are far-reaching decisions, he will just stop by later to visit you”. (R28).*

Participants who did not need **support for themselves**, could easily understand that **for others counselling would be helpful**, for example, when there is no agreement between relatives, when people are incompetent to decide because of a shock, when they experience little support from their relatives, or when the wish of the deceased is unknown. The **kind of support** mentioned was mediation (when there is disagreement between relatives), creating a pause for reflection in a crisis, giving information and explanation about organ donation, being a coach in decision-making or during emotional reactions. This task could be attributed to different **professions** (Transplant Coordinators, Social Workers, Psychologists, Hospital Chaplains), acting as a confidant.

#### Discussion

The discrepancy between general willingness to donate and the actual refusal of a donation request can be explained by multiple factors, with a cumulative effect.

Firstly, half of the participants (most non-donor families) stated that they felt that they were not competent to decide in such a crisis, which is confirmed by other research [6]. The emotional crisis might lead to being unable to think of sufficient ethical considerations. The participants of this study reproduced far fewer considerations in their crisis than the respondents in ‘normal’ circumstances in the studies of Newton [18]. Furthermore, they were confronted with contradictory considerations *pro* or *contra*. Many considerations mentioned by the participants were similar to a number of ‘beliefs’ in the meta-study of Newton [18], but could lead to ambivalence. Just as Sque, it was discovered that relatives can struggle with utilitarian considerations against their wish to protect the body [4, 19, 20]; for non-donor families in the sample, this could lead to an unresolved dilemma. In contrast to Newton’s study, the ‘integrity’ consideration was not religiously founded, perhaps, because most Dutch people do not experience their spirituality in an institutional way [21]. Religion was not mentioned by our participants as influential. Another difference with Newton’s study is that the interviewees in this study never used mistrust of the medical profession as a consideration.

To overcome the incompetence to decide, it is suggested to wait with the donation request to give relatives some time to accept the death of their family member. Thus, this study underscores the importance of decoupling, which is advised by literature [10, 22].

Secondly, the majority of non-donor families did not know the deceased’s wishes. Bramstedt et al. reported that when the wish of the deceased is known, families feel themselves ethically obliged to make a decision that represents the values and preferences of those whom they represent [23]. Indeed, the donor families in this study did not report much distress in decision-making, when they could honour the expressed preference of their deceased. However, non-donor families felt distress, because they were –in their opinion– the heirs of the body and had the deciding vote. These non-donor families thought they had more right to decide about the body in their own way, because they had to live with the decision. Thus, considering the decision as a surrogate decision [23] –which is also the intention of the Dutch Law on Organ Donation– might apply to donor families but not to non-donor families. Posthumous autonomy is contested by non-donor families. When forced to make a surrogate decision, some non-donor families refused consent for donation, because their deceased had not given any directive. That confirms findings that state that families feel ‘left in the dark’ when they have to decide on behalf of the deceased person [5, 23]. Helping relatives to remember what the deceased would have wanted, elucidate prejudices and enumerate considerations might support a well-considered decision [24]. This approach seems worth investigation.

Thirdly, the findings emphasise the importance of Donor Registration, because it seems to prevent dilemmas in decision-making, at least for donor families. Nearly all donor families had a hold on the registration in de National Donor Register. Relatives of deceased, who had not registered in the National Donor Register, were more inclined to remain ambivalent in their decision. Their dilemma could not be resolved by weighing values and convictions. In these cases, they justified their decision with arguments derived from the context (lack of time, information etc.). Non-donor families did not regret their decision, but half of them remarked that their decision could have been different, provided that the context had been different. This illustrates the instability of the decision, an instability that was also found in other research [9–13]. To resolve their dilemma, a form of support might be desirable. Half of non-donor families would have accepted a form of (extra) support, whereas nearly all donor families would have declined it. A long-term contact as suggested by Aldridge [22] in combination with a well-trained donation professional can stimulate the family to decide in a well-considered way and might have a positive impact on the family consent rate [25].

As separate point, refusal for consent was mostly defended by various considerations against a complete or a partial consent for DBD. The duration of the donation procedure and/or the exclusion of specific organs (especially heart) and/or the wish to be present at the moment of visible death were, for some non-donor families, a compelling reason to refuse donation, and for three out of the twelve donor families to agree only with a DCD-procedure, although a DBD was possible. In this study, a DCD was not suggested to any of the non-donor families who refused to give consent. Offering the possibility of DCD (when possible), in case the relatives refuse consent of DBD, might facilitate consent in a subgroup of non-donor families, when relatives have objections because of the time needed for a donation procedure, the wish to be present at the visible death, or the exclusion of organs such as the heart.

### Strength and constraints

This study adds a new perspective to the literature on decision-making on organ donation, as many non-donor families were interviewed. Although the sample was not representative, a qualitative comparison of donor families and non-donor families could be made to provide a unique insight into the reasons why non-donor families refuse consent for organ donation. The study was retrospective and explorative. To confirm the suggestions made, further research (an intervention study) is required.

## Conclusions

Discrepancies between willingness to consent to donate and refusal at the bedside can be attributed to an unresolved dilemma: aiding people or protect the body of the deceased. Non donor families feel incompetence to decide and refused donation, whilst their deceased had not given any directive. When ethical considerations do not lead to an unambiguous answer, situational factors were pivotal. Relatives of unregistered eligible donors are more prone to unstable decisions. To overcome long term ambivalence, coaching during decision-making is worth investigation.

### Ethical considerations

The protocol was approved by the Institutional Review Board of the Radboud university medical center, Nijmegen the Netherlands (permission was given with number ABR nr NL.21205.091.07 d.d.10 June 2008) before the study commenced and later by the Institutional Review Board of the Sint Elisabeth Hospital, Tilburg the Netherlands (permission was given with protocol number 1106 d.d.25 March 2011) to get additional data.

All participants gave their written consent before being included in the study.

### Availability of data and materials

Not applicable.

## Abbreviations

DCD: Donation after circulatory death
DBD: Donation after brain death
